# Supplementary figures and images for: Application of High Resolution Melt analysis (HRM) for screening haplotype variation in a non-model plant genus: Cyclopia (Honeybush)
Source: PeerJ. 2020 May 15;8:e9187. doi: 10.7717/peerj.9187 (PMC7233275; doi:10.7717/peerj.9187)

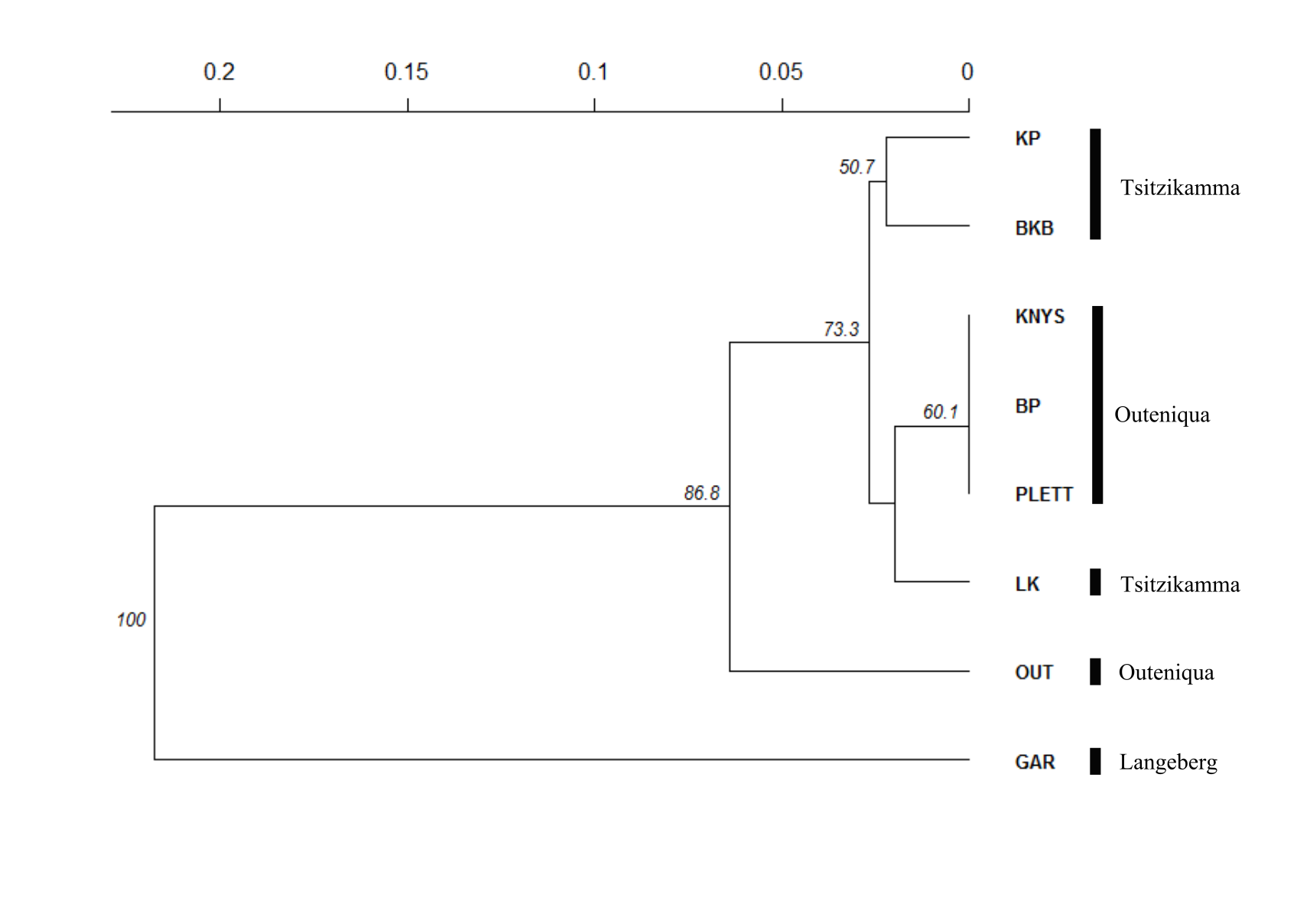

Supplement: Fig. S5 [file peerj-08-9187-s005.png]
