# Supplementary material for: Application of High Resolution Melt analysis (HRM) for screening haplotype variation in a non-model plant genus: Cyclopia (Honeybush)
Source: PeerJ. 2020 May 15;8:e9187. doi: 10.7717/peerj.9187 (PMC7233275; doi:10.7717/peerj.9187)
Supplement: Supplementary file 4 [file peerj-08-9187-s002.doc]

Primer origin	HRM Primer	PCR product size (bp)	Sequence (5'->3')	Tm	GC%
trnG intron	MLT_C1	150	ACTCCTCTTCTATTCATGGGGA	57.3	43.5
	MLT_C2		TTCATTCGGCTCCTTACGG	58.0	50.0
	MLT_C3	236	TCAACGAACGATTCGAGGAATA	57.6	40.9
	MLT_C4		TGCTTCAATCTCTCCTACCCAA	58.8	45.5
	MLT_C7	*419*	*AATCTTTTATACCTCTCAACGAACG*	*57.5*	*36.0*
	MLT_C8		*TCCCCATAGAATAGAAGAGGAGT*	*57.3*	*43.5*
pctL-psbE intergenic spacer	MLT_M1	170	TGTCGAGAACCCTTATACTCTCA	58.2	43.5
	MLT_M2		TACCAAGGGTGTCTTTCGAGT	58.7	47.6
	MLT_M3	*540*	*TCAATTCATCATCTGTGATTCCAAA*	*57.3*	*32.0*
	MLT_M4		*GGTTCTCGACATTATTAGCTTCGG*	*59.6*	*45.8*
atpI-atpH intergenic spacer	**MLT_S1**	**217**	**TGGGGGTTTCAAAGCAAAGG**	**58.9**	**50.0**
	**MLT_S2**		**ATTACAGATGAAACGGAAGGGC**	**58.7**	**45.5**
	**MLT_S3**	**310**	**TTCCCGTTTCATTCATTCACATTCA**	**59.8**	**36.0**
	**MLT_S4**		**CCTTTGCTTTGAAACCCCCA**	**58.9**	**50.0**
psbD-trnT intergenic spacer	*MLT_R1*	*150*	*GGTTTGGACTCCCTAATAATCTGTC*	*58.9*	*44.0*
	MLT_R2		*ATGTTAGAACTTGGAATAAGCCCT*	*57.9*	*37.5*
trnQ-5'rps16 intergenic spacer	*MLT_T1*	*493*	*TCCTTTAGTTTGGATCCGGTATGT*	*59.5*	*41.7*
	MLT_T2		*GGGCCAAGGGTAAGGCAA*	*59.6*	*61.1*
ndhA intron	**MLT_U1**	**345**	**AGGTACTTCTGAATTGATCTCATCC**	**58.2**	**40.0**
	**MLT_U2**		**GCAGTACTCCCCACAATTCCA**	**60.0**	**52.4**
rpl32-trnL Intergenic spacer	MLT_V1	340	CTCCTTCCCTAAGAGCAGCG	59.9	60.0
	MLT_V2		GTTGGAATAATCTGAATTAGCCGGA	59.2	40.0
