# Supplementary material for: Application of High Resolution Melt analysis (HRM) for screening haplotype variation in a non-model plant genus: Cyclopia (Honeybush)
Source: PeerJ. 2020 May 15;8:e9187. doi: 10.7717/peerj.9187 (PMC7233275; doi:10.7717/peerj.9187)
Supplement: Supplementary file 5 [file peerj-08-9187-s003.doc]

	ndhA intron								*atpI-atpH* intergenic spacer		
Haplotype	104	183	184	185	186	187	188	189	349	380	381
A	c	g	g	g	g	a	t	a	c	t	c
B	t	-	-	-	-	-	-	-	c	t	c
C	c	g	g	g	g	a	t	a	t	t	c
D	c	g	g	g	g	a	t	a	a	t	c
E	c	g	g	g	g	a	t	a	c	a	a
