# Supplementary material for: Application of High Resolution Melt analysis (HRM) for screening haplotype variation in a non-model plant genus: Cyclopia (Honeybush)
Source: PeerJ. 2020 May 15;8:e9187. doi: 10.7717/peerj.9187 (PMC7233275; doi:10.7717/peerj.9187)
Supplement: Supplementary file 6 [file peerj-08-9187-s004.doc]

AMOVA results obtained from testing structure across mountain ranges

	Degree freedom	Sum Sq.	Mean Sq.
Between samples	3	84.1	28
Within samples	138	39.5	0.3
Total	142	123.6	0.9

Components of covariance

	Sigma	%
Variations Between samples	0.8	73.8
Variations Within samples	0.3	26.2
Total variations	1.1	100
